# Efficacy of dietary interventions in end-stage renal disease patients; a systematic review

**Published:** 2015-10-01

**Authors:** Chaudhary Muhamamd Juniad Nazar, Micheal Mauton Bojerenu, Muhammad Safdar, Armughan Ahmed, Muhammad Hammad Akhtar, Tiffany Billmeier Kindratt

**Affiliations:** ^1^Department of Nephrology, Shifa International Hospital, Islamabad, Pakistan; ^2^Department of Internal Medicine, Sickle Cell Unit, Harvard University Hospital, Washington DC, USA; ^3^Department of Nephrology, Pakistan Institute of Medical Sciences, Pakistan; ^4^Department of Family and Community Medicine, University of Texas Southwestern Medical Center, Dallas, USA

**Keywords:** Cardiovascular disease, Chronic kidney disease, End-stage renal disease, Diabetes mellitus, Diabetic nephropathy, Diet

## Abstract

Cardiovascular disease (CVD) and chronic kidney disease (CKD) are common comorbid conditions. Life style, particularly diet is a critical component of treatment for these conditions. Register dietitians play a key role in bridging the gap between the science of nutrition and the empowerment of individuals to alter their lifestyles in a healthy manner. A range of dietary manipulations has been reported to reduce risk factors and decrease risk of CVD and CKD outcomes. However, many studies provided food to participants or were limited to adjustment of few specific nutrients. Diet intervention in relation with end-stage renal disease (ESRD) is really complicated topic. As multiple co morbid conditions such as hypertension, CVD, CKD, and diabetes mellitus (DM) are associated with ESRD, which made the scenario really worse while fixing the dose of any diet. Still a lot of research work is required to understand this topic.

**Implication for health policy/practice/research/medical education:**

Cardiovascular disease (CVD) and chronic kidney disease (CKD) are common comorbid conditions. Life style, particularly diet is a critical component of treatment for these conditions. Register dietitians play a key role in bridging the gap between the science of nutrition and the empowerment of individuals to alter their lifestyle in a healthy manner. A range of dietary manipulations has been reported to reduce risk factors and decrease risk of CVD and CKD outcomes.

## Introduction

Helping patients with end-stage renal disease (ESRD) attains or maintains the best nutritional status possible is difficult. Even for patients who are on dialysis for years, maintaining the necessary balance of various nutritious is arduous. The addition of any other chronic disease only adds to the challenge. Approximately one third of patents on dialysis have ESRD secondary to diabetic nephropathy.

## Methodology

### Study design

Systematic review is the rigorous reviews of specific clinical questions. It is widely disseminated and has implemented on health care policy and practice, both in the united States and internationally (1). It is known as systematic because of its design to summarise the original research bearing on the question following a scientifically based plan that has been decided in advance and made explicit at every step. Clinical decisions are based on the weight of evidence bearing on a question. Systematic reviews are specifically useful in addressing the single focused question. Sometimes, large studies are so compelling that they eclipse all studies that preceded them. More often, however, clinician depends on the accumulation of evidence from many less definitive studies. Conventionally, systematic reviews are needed to establish clinical and cost-effectiveness of an intervention or drug. Increasingly, however, they are required to establish if an intervention or activity is feasible, if it is appropriate (ethically or culturally) or if it relates to evidence of experiences, values, thoughts or beliefs of clients and their relatives (2). Systematic reviews are surveys of research articles, instead of people (3). We selected the systematic review as our study design for postgraduate thesis while, it collates all empirical evidence that fits pre-specified eligibility to answer a specific research question. The question of research project was more clinical and a lot of research has already been done with different research design. It included several primary studies – perhaps with disparate findings – and substantial uncertainty. The systematic review offered me different advantages, which made me eager to select it, including comparison of different studies research design from different environment, it is significant in making good and precise decision by studying different trends and relationships in causing factors but as precise estimates of the interventions affect and it has a good quality control, and its statistical power is excellent (4).

We carried out systematic on this area because we have found that there is huge gap and very less work has been performed on the effects on dietary intervention on ESRD patients. We tried to identify observational or interventional trials assessing effects of diet on the pathogenesis of ESRD and though to study them all together to have more variable results. It is basically a trial to inform or educate renal practitioner about the importance of diet in ESRD patients based on new literature published. There was a lot of research work published in area of interest that made it difficult and challenging for clinician to stay informed for the latest medical development and to maintain current evidence based knowledge. Nutrition in ESRD was a complex and confused topic especially due to associated co morbidities. We also intended to purpose a future research agenda as still we felt the question somewhere left unclear due to its sensitivity and complex nature. Systematic reviews needed to propose a future research agenda (5) when the existing agenda have failed to address a clinical problem. Moreover, we selected systematic review as our research design due to lack of medical resources, shortage of time and it is easy in approach. We believed, it was chance to read a lot of medical literature based on research question in a short time. Even we hoped that my systematic review helped clarify a vital issue to the public and to healthcare professionals; preparing such a review, however, is not a trivial exercise.

### Data source and search strategy

We developed a search strategy to investigate my research question by searching in Medline and grey literature. Grey literature included abstracts presented in the years 2000 through 2010 at the annual meetings of the American Society of Nephrology, National Kidney Foundation, and European Renal Association and the websites.

#### Computerise literature

- Databases of pre-appraisal articles
- American College of Physicians PIER
- BMJ point-of-care
- Clinical Evidence Cochrane database of systematic reviews
- Database of Abstracts of Reviews of Effectiveness (DARE)

#### Databases of synthesized evidence

- Clinical trials
- NHS Economic Evaluation

The following key words and MESH terms were used: “kidney disease,” “comorbid condition,” “dialysis,” “end stage renal failure,” and “transplant.” The terms were used in combination also ‘chronic kidney diseases + nutrition’, or ‘renal transplantation + diet’ and ‘ESRD’ + ‘diet’ was combined with the terms prognosis, mortality, outcomes and diagnosis.

We reviewed the bibliographies of relevant studies, the “Web of Knowledge Cited References” list, and the “Related Articles” link in PubMed to identify additional studies. We also use key words such as diet intervention, “lipids,” “proteins,” and “carbohydrates.”

We selected articles, which were published in English. The articles other than English were not included. To avoid duplicate publications, the most recently published version is used. Further attempts were made to trace unpublished or missed studies (grey literature). We also searched the other data available online or by university of Brighton library available.

### Inclusion criteria

- An observational study or a randomized controlled trial (RCT) aimed to analyse the impact of diet modification, smoking, obesity in ESRD patients.
- Studies that analyzed the different condition such as hypertension, diabetes, CVDs and their impact of renal replacement therapy.
- The articles published between 2000 and 2010 were included.
- Studies published in English language.

### Exclusion criteria

- Case reports and case series
- Studies that used low-protein diets
- Studies that analysed the role of other factors such as weight loss, other diseased condition including stroke, neurological conditions in dialysis patients or CKD were excluded also.
- We excluded duplicate studies published on same set of patients. The articles published before 2000 were excluded.
- The studies from other under developing countries were not involved because quiet less research work was found; articles related with the primary health care for CKD are excluded as our research is focused on impact of nutrition on the ESRD patients.

### Types of participants

The types of the participants involved in the studies were diagnosed patients of CKD, who were at different stages and its associated co-morbidities such as diabetes, CVDs, and hypertension and also patients on renal replacement therapy. Basically, all the patients with ESRD on were maintained on dialysis or have kidney transplant were eligible irrespective of age, sex, race or primary renal disease co morbidity. Their involvement has strong influence on the intervention strategies for the control of CKD prevalence. The number of participants ranged from the 155-140 000 mostly from age 55-68. The patients involved in the studies were considered regardless of their race or ethnicity mostly residing in developed countries. The patients who their first language, were not English but if they can understand or speak English were also included. The studies in which patients had neurological condition were not included. Trials that exclusively comprised patients with acute renal failure were excluded. Each individual study’s definition of ESRD or maintenance hemodialysis was accepted.

### Data extraction and quality assessment

Data extraction is defined as the scale at which it employs to measure, to reduce the bias and error in its design, conduct and analysis (6). The quality of included studies was assessed initially by author and reviewed independently by the academic supervisor without blinding to authorship or journal using the checklist developed. Any discrepancies were resolved by discussion.

After applying inclusion and exclusion criteria, we were left with studies having different research designs. We performed systematic review by involving different methodology that cross-sectional studies, literature review, randomized controlled trial and descriptive study because it preserve the integrity of the findings of different types of studies by using the appropriate type of analysis that is specific to each type of finding.

### Study selection

We considered all the effective steps in selecting the articles in order to avoid publication as well as selection bias. We involved four reviewers with me. Out of which two are used in covering the name of the article and authors’ name, the two reviewers involved in the study reads the journals carefully stating with the title then followed by reading the abstract that are related to the topic under study to see if they match to the purpose of the research. Based on research question, 10 articles were found to meet the inclusion criteria and also are of good quality. These 10 articles were carefully read and analyzed by the reviewers. Two reviewers did the data analysis and data was synthesized according after agreement and disagreement.

The quality items to be assessed will be evaluated by following elements; each element will be assessed separately rather than combined in a scoring system.

- The article must be published in the journal.
- Researcher must be experienced and qualified to perform the research work.
- The way of selection of subject selection must be checked or if it is article then the inclusion and exclusion criteria will be considered.
- Description of therapeutic intervention.
- Description of subjects not completing the study.
- Acceptable subject completion rates.
- Appropriate measurements about confidentiality and other factor, which can produce bias in the studies.
- Data analysis.
- Information on adverse effects.

When multiple publications from the same group were found, the studies were reviewed carefully to ensure that no data were analyzed in duplicate. Data were extracted into a database and checked for accuracy by a second reviewer. When data were reported in strata, the data were extracted as separate cohorts. The following data were extracted from each included study: design, definition of CKD, age, gender, baseline renal function, clinical subpopulation (heart failure, known CVD, hypertension, general population, and other), cardiovascular risk factors other than CKD (hypertension, smoking, diabetes, blood pressure, low-density lipoprotein cholesterol [LDL-C] and body mass index [BMI]), proteinuria, medication use (angiotensin-converting enzyme inhibitors, aspirin, beta blockers, and statins), and mortality (all-cause and cardiovascular). We developed a data extraction sheet. One author extracted the data from included studies, and the second author checked the extracted data. Disagreements were resolved by discussion between the two review authors.

### Data synthesis

This systematic review involves studies with different study design, methodology and findings. Each research work was reviewed completely. The summary was prepared for the purpose comparison with other studies. The summary of the finding from the research was incorporated and interpreted into a comprehensive conclusion. The eligibility of each citation was evaluated and the full-text article of each citation was retrieved for any citation considered potentially relevant. Two reviewers independently screened the citations and those considered potentially relevant were retrieved for full-text review. We independently evaluated the eligibility of each full-text article, with disagreements resolved by consensus with second reviewer.

## Results

### Search yield

**Figure 1:**
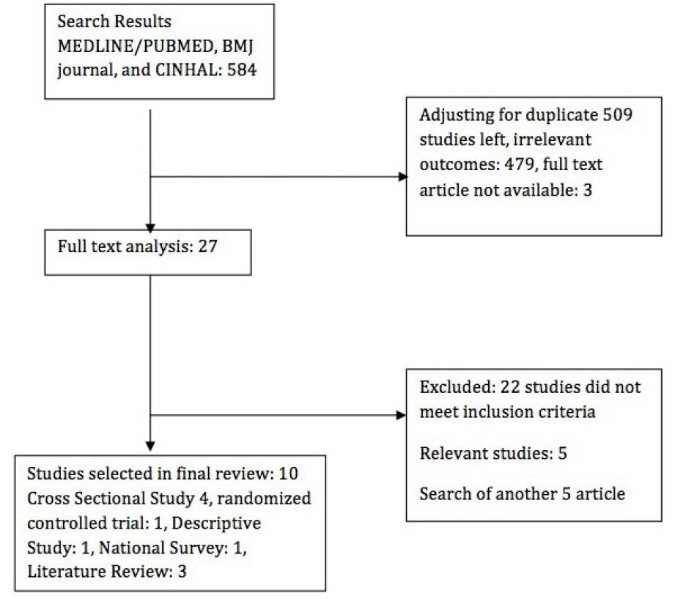
Flow chart showing the search, the major reason for the exclusion of studies and the final number of the article included in the studies

Figure 1 summarizes the search yield. No articles examined blood pressure targets exclusively in patients with CKD and diabetes.

One of the 10 studies was nested cohort analysis of randomized, controlled trials. Of the remaining 8 included studies, 3 were literature review, 4 were cross-sectional trials, 1 was a descriptive study cohort study, and 1 was a national survey. The sample selection process and the prognostic variable were described adequately in 100% of the studies. As well, most (97%) described the inclusion and exclusion criteria adequately. Other quality items such as the description of clinical and demographic characteristics, the description of outcomes, and adjustment for important prognostic factors were adequate in 74% to 90% of the studies. In all studies, the effect of nutrition on ESRD patients was discussed. Studies also focused on different scenarios such as malnourished patients in renal replacement therapy along with different risk factors, which make the prognosis of ESRD in relationship with nutrition. The strength of the research work is that in all studies, the definition of CKD seemed to be based on not on single measurement of serum creatinine but they also used anthropometric measurement to confirm diagnosis and avoid the bias. The number of patients who were lost to follow-up was reported in 67% of the studies in national survey and cross sectional study. Of these 81% of studies lost <10% of patients, during their follow-up.

### Outcome measures

#### 1. Relation between CKD and nutrition

This systematic review was designed to examine the effects of nutrition on the pathogenesis of the ESRD and determine whether it can enhance or impede the progression of disease. There are some studies favouring the main outcome and also few studies, which are highlighting factors having a potential role on the pathogenesis of ESRD. A cross-sectional observational study performed by Nair in which they investigated 27 patients with advanced chronic renal failure (CRF) on their first visit highlight the patients with advanced CRF without specific treatment, evaluated for the first time, showed fat store depletion. They evaluated the patients by anthropometric patients. They found that all the patients were malnourished (7). Similarly study performed by Motokawa et al. At tertiary hospital pre-dialysis out-patients clinic by involving 56 consecutive patients with CKD showed that the prevalence of malnutrition among these was huge whether they are on dialysis or with kidney transplant which make the disease worse (8).

Nutritional supplementations in any form intradialytic parenteral nutrition (IDPN), daily oral dietary assistance supplementation and intradialytic oral nutrition (IDON) increase the serum albumin concentration in ESRD. Nutritional intervention that increase the serum albumin may lead to considerable improvements in the mortality, hospitalization, and treatment costs (9). Similarly, study highlight that the importance of protein diet and amino acid, which have very important role in the metabolism of kidney and showed that if diet is improved it can impede the progression of CKD to ESRD (10).

#### 2. Different risk factor effecting nutrition in ESRD

Selected studies in systematic review also highlight the facts for example late referral of patients, older age, and the existence of multiple co-morbidities, lack of education and awareness among ethnic minorities, communication barrier between primary health care professional, and the shortage of the multidisciplinary care team for dialysis centers was the associated characteristic with the increasing prevalence of ESRD. Most studies also identified multiple factors such as drug-drug interaction during the treatment, lack of anemia management during dialysis and hypertension, depression in the patients of CKD that is poor quality of life during the treatment as a risk factor enhancing the progression of disease. Selected studies also highlight the comorbid conditions such as increasing frequency of obesity, hypertension, diabetes, CVD, regional difference in prevalence of ESRD, and nutrition practices done by registered renal dietitian as a risk factors which effect the progression of disease (11,12).

## Discussion and Conclusion

In the section, discussion has been carried out by article obtained in search work and comparing them with the previous work done in area of the interest. It also highlights the barriers face by the author while performing the research work. Discussion has been carried according to the clinical outcomes of the study.

### Outcomes related with diets

Helping patients with ESRD attains or maintains the best nutritional status possible is difficult. Even for patients who are on dialysis for years, maintaining the necessary balance of various nutritious is arduous. The addition of any other chronic disease only adds to the challenge. Approximately one third of patents on dialysis have ESRD secondary to diabetic nephropathy (12). Study performed in Japan found the disparity between occurrences of ESRD throughout the country. Generally speaking the people of Japan are racially homogeneous. Although they believed that there are a factor other than genetic which contribute to the occurrence of ESRD, these risk factor include obesity, dyslipidemia, hypertension, diabetes mellitus (DM), osteopenia, impaired growth in children, protein –calorie malnutrition (muscle wasting syndrome), micronutrient malnutrition, mineral and electrolyte malnutrition, and anemia*.*

Increasing frequency of obesity, hypertension, and diabetes threaten disturbing trends signaling an ever-increasing epidemic of both CVD and CKD. Management of CKD and CVD should include both medication and nutritional therapy. Medical nutrition occupies a critical role in optimal treatment of these conditions. Once CKD is identified action should be taken to prevent progression of kidney disease as well as to reduce CVD risk. Numerous interventional therapies have been proposed to reduce risk factors, slow progression of CKD and to reduce mortality and CVD events (13). Intensive dietary intervention with a low fat, low salt, and low protein diet was recognized as an effective approach to modify pathological processes that result in hypertension, diabetes, CKD and CVD. Such strategies, although requiring dedication to fairly extreme lifestyle change, remain effective therapy.

### Hypertension

Worth of nutritional intervention for controlling blood pressure is well authenticated. Packard et al (9) showed that nutritional therapy occupies a very important role in reducing preventable risk factor such as hypertension and DM and prevent damages to the kidneys. There are many dietary regimens that claim to provide health benefits but on long-term basis efficacy is frequently lacking (13). Study was performed to check out different dietary approaches to stop hypertension. The different diets included were low fat dairy foods, and limiting total fat, saturated fat, and cholesterol. Observance was associated with decrease blood pressure in this landmark study (14). Similarly, a route of the same eating style augmented with sodium limitations was performed by another study. The study indicated decrease in blood pressure that was intensified, as sodium intake was reduced step wise from 140-100 to 65 mmol/day (15). Because use of high sodium may contribute to progression of kidney disease, especially in the early stages; this eating style may offer benefit for the ESRD patients (16). Recently, addition of soy protein or predominantly vegetable protein to the diet was shown to lower blood pressure in person with pre hypertension or untreated stage 1 hypertension (17,18). Nutritional therapies for controlling blood pressure are still questionable. Therefore, a lot of research is needed to continue to explore the best nutritional therapy to control blood pressure.

### Diabetes mellitus

Nutritional therapies are a cornerstone in diabetes management and are critical to achieving goals for glycemic control (19). Optimal nutritional support in ESRD is not well defined and can be provided at different levels. A study by Appel et al performed in the United States, indicated that 43% of HD patients have diabetes because most dialysis patients are not fasting when blood samples are drown. In 2005, according to US Renal Data System, more than 20% patients received no HbA1c testing. HbA1c is a blood test used to detect glucose in diabetes patients. Results from the present study, in which respondents indicated only 11% of the patients did not receive testing, suggests that routine assessment of glycemic control may be increasing (20). Studies have shown that complications associated with DM can be reduced by intensive glycemic control. This intensive control can help to reduce the severity of CKD and CVD as well (21). A recent prospective study was performed in the United States with the help of renal dieticians. Registered dietician’s counseled renal patients to follow American Diabetes Association type diet, which emphasize to improve the glucose level by reducing weight and balance diet by control of calories and carbohydrates intake as well as reducing dietary saturated fat and cholesterol. Study showed the benefits of nutritional counseling on the health status of adults with type 2 diabetes (22).

### Cardiovascular disease

Among nutritional therapies devised for reducing CVD risk, a portfolio modifications has emphasized very low saturated fat intake and included plant sterols, soy protein, viscous fibers from grains and vegetables, and salts. This nature of diet has been related with substantial drop in low-density lipoprotein cholesterol (LDL-C) and C-reactive protein to levels similar to those seen in patients treated with drugs therapy (23). However, this dietary regimen requires intake of large amount of foods uncommon in Western eating patterns, including soy protein sources, a migraine product containing plant sterol and foods high in soluble fiber such as oat, bran, psyllium, barley, eggplant, and okra. Therefore, instead of taking fast food always other balance diets must be tried to have a good clinical outcomes.

Most dialysis center use anti-hypertensive drugs and some use low salt diet to control the blood pressure or hypertension in dialysis patients. The cross-sectional was performed to assess the benefit of salt restriction in hemodialysis patients. The study indicated that reduced salt use and reduced prescription of antihypertensive drugs can result in reduce cardiovascular events for example it will preserve left ventricular function, reduce intradialytic hypotension in hemodialysis patients (24). Ornish et al showed significant regression of coronary atherosclerosis associated with a very low fat (10% of calories) high carbohydrates (70% to 75% of calories) diet nearly devoid of animal foods. Intensive physiological support characterized this in addition to diet (25). Although these studies indicate substantial benefit derived from prescribed diet and life style changes, compliance requires extensive departure from the predominant culture.

### Obesity

Obesity has been the subject of increasing scrutiny in the recent years as a risk factor for poor outcome in transplant recipients, especially renal transplant recipients. Over 20% of renal transplant recipients become obese after a successful transplant and BMI >22 kg/m^2^ is associated with increased morbidity. When considering that adverse impact of metabolic syndrome on outcome, most likely due to pre-transplant nutrition and cachexia (26). In the renal transplant recipient, would dehiscence and infection have been reported with increased frequency, whereas infection of all type occurs more common in obese heart and live transplant recipients (27). Although initially counterintuitive, there are little data to back up the contention that pre-transplant weight loss protocols will lead to better outcome (28). In fact, renal transplant recipients with documented weight loss during the time they are on the waiting list demonstrate faster weight gain post-transplant (29,30). One research note, studies in the pre-transplant period should use nuclear magnetic resonance techniques for the determination of total body potassium as more accurate marker of increased body cell mass and dual energy x-ray absorptiometry to assess body composition (31,32). Post-transplant weight gains tend to be progressive is associated with the development of other features of post-transplant metabolic syndrome, and is severely impacted by the glucocorticoids dose used and more rapid glucocorticoids withdrawals protocols (33,34).

### Proteins

In recent years, high protein diets have been promoted as weight loss strategies (35). Lack of currently expected definition for high protein or low carbohydrate diet can make comparison and evaluation of diet difficult. Recommended intake for macronutrients vary widely as reflected in the release from the Institute of Medicine, with calorie distribution of 45% to 65% from carbohydrates, 20% to 35% from fat and 10% to 35% from protein (36). Study performed by Haggan et al indicated that disparity of prevalence of occurrence of ESRD is due to the lack of proper diet. It is due to restriction of protein. It is proved by animal models that diet rich in protein can stop progression of different nephrology disease. However is not proved by large-scale clinical trials yet (37). Unfortunately, the studies performed to evaluate high protein loss for weight loss enrolled a small number of healthy subjects (38). They performed evaluated high protein diet as strategy for weight loss. None has adequately tried to evaluate the potential risks or effect on CKD or CVD. On the other hand, balanced low calorie diets are considered as the most weight loss strategies over a long duration (39). In a recent review, Kennedy et al (40) concluded that high protein diets are contraindicated for persons with CKD. High protein intake has been shown to increase the risk of micro-albuminuria in hypertensive diabetic patients (41). Furthermore, women with mild renal insufficiency (estimated GFR 55 TO 80 mL/min per 1.73 m) who consumed more protein had a 3.5-fold greater risk of losing kidney function over 11 years compared with those who consumed less (61 g/day). Interestingly, the risk of higher protein intake seemed to be most related to consumption of meat. When non-diary animal dairy and vegetable protein intake were examined, non-diary animal protein was associated with the great decline in estimated glomerular filtration rate (GFR) (42).

Adverse effects of excess dietary protein are biologically plausible. Glomerular hyperfiltration and hypertension, major mechanism of kidney injury, are produced by high protein diet (43). These effects are particularly pronounced in those with diabetes (44,45). Recent data indicate that a level of amino acid similar to that in blood after protein meal induces changes associated with injury in glomerular mesangial cells (46). At the other end of the spectrum, dietary protein restriction was effective in slowing progression of CKD (increasing albuminuria/proteinuria or decrease in GFR) in meta-analysis of 10 studies on diabetic and non-diabetic renal disease. Most importantly, a modest limitation of dietary protein (0.89/kg body weight /day versus 1.02 g/kg body weight /day) greatly reduced risk for ESRD or death risk (47).

Although evidence is insufficient to assume harm to individual without CKD, long-term adherence to a high protein diet carries potential for deleterious effects. Evaluation of kidney function of any individual is recommended before initiation of a high protein diet (48). This should include the testing of microalbuminuria or macroalbuminuria and estimate of kidney function based on the guideline set by the National Kidney Foundation (49). High protein diets are not advised for persons with albuminuria levels above 30 mg/g or GFR below 60 ml/min/1.73 m^2^ because of their increased vulnerability to adverse effects on the kidney. Based on evidence available, nutritional interventions for CKD that limit dietary protein to 0.6 to 0.8 g/kg/day depending on the stage of disease are prudent (50). Although hypertension studies suggest that higher protein intake may improve blood pressure control, these benefits seem to be confined to vegetable protein. Vegetable protein may also be renal sparing. Therefore, a higher protein intake that emphasizes vegetable source may be a reasonable approach in hypertensive patients without CKD (51).

### Protein calorie malnutrition

Up to 15 % of renal transplant recipient may display signs of malnutrition (52). A cross-sectional survey performed by Hussaini et al by enrolling 27 patients (13 women) with advanced CRF on the occasion of their first visit at Nephrology Division of University Hospital. The results indicated that patients were malnourished at their first visit. The fat stores were depleted in visiting patients. Depletion of fat was not linked due to the inflammation or other visceral protein loss. It was linked with low energy intake. Therefore, the specialist must follow CRF patients from the very beginning (53). A transplant recipient whether of kidneys or liver may experience, a constant weight reduction in body. Muscle wasting, weight loss, and micronutrients deficiencies of vitamins B-complex have been also reported at different stages of transplantation. Protein calorie malnutrition (PCM) may occur in the early postoperative weeks when glucocorticoids do are highest and the protein catabolic rate (PCR) is augmented due to surgery or rejection (54). Low BMI is considered as the risk factor for death and pre-transplant cachexia in both kidney and heart transplant recipients (55,56). During the 3 to 4 post-transplant week or whenever high dose of steroids for acute rejection, a diet composed of high protein is recommended (1.5 to 2.0 g/kg/day) (57).

### Potassium

A small majority reported using 5.5 mmol/l, as the acceptable upper limit for serum potassium, but a substantial minority reported using 6.0-mmol/l. Hyperkalemia is responsible for 3% to 5% of deaths in dialysis patients (58), yet none of the KDOQ1 guidelines address potassium, and the little consensus excited among the 11 references that did. Five studies recommended 5.5 mmol/l (59). A large retrospective, observational study recommended 5.3 mmol/l (5.3 mEq/l), as the upper limit (60). Three equated a serum potassium (K) level of >6.0 mmol/l (0.6 m Eq/l) with hyperkalemia (61). However, the measurement assessment tool to which the immunoglobulin (IG) refers respiratory distress syndrome (RDS) does not include target ranges for serum potassium (K). Nearly half the dieticians in this study stated they would like to see the development of a clinical practice guideline on potassium.

### Vitamins

A cross-sectional study was performed in the United States, in which 1270 renal dietitian were invited. The study highlighted that 95% of the renal patients were advised to take vitamin supplementation but patients do not take renal vitamins supplements regularly as they were prescribed (62). Folic acid is the most commonly recommended renal vitamin supplement by renal dieticians. Most renal dieticians recommend a dose of 1 mg/day (63). But few dieticians did not agree with them and they advised their patients to use folic acid in dose of 1-10 mg including some other vitamin supplements (64). On the other hand, few studies suggested that folic acid is not so compulsory. But few agree to take folic acid in combination with other supplementation (65). However, the interesting thing noted was that the KDOQL nutrition guidelines do not address the issue of vitamins supplement dosages in dialysis patients. The Dialysis Outcome and Practice Pattern Study (DOPPS) data indicate that patients who took a multivitamins had a 16% lower mortality risk compared with non-vitamin takers (66). The survey indicated that 80% of renal dieticians agreed that guideline must be developed for vitamins supplementation. Vitamins requirements for dialysis patients represent an enormous area for future research.

### Nutrition in transplantation

Literature review carried out by Harry (11) indicated that malnutrition is very common among kidney transplant patients. Especially, after transplant complications increases due to the use of immunosuppressive drugs which lower the immunity of the patients. After the early transplant years, infection and rejection are the major risk factor that recipient faces. Advances in immunosuppressive therapy have prolonged the life of transplant but little has been done in terms of dietary, nutritional or bio behavioral modification. In order to make transplant successful clinical research efforts using interventional dietary trial are lacking and must be performed (67). The greatest need for nutritional research in transplantation focuses on the lack of interventional trials to eliminate over nutrition, reduce weight pain, and mitigate the effects of early post-transplant metabolic syndrome. Only handful of short term studies, usually in renal transplant recipients and encompassing only about 1 year in duration, have been published to date documenting an improvement in post-transplant metabolic syndrome.

Bellingheri and colleagues recently reported improvement in post-renal transplant BMI and metabolic syndrome during 1 year of dietary intervention (68). These investigators showed that low fat, hypocaloric diet could lead to steady reduction in BMI while steroids were being tapered. In another European dietary intervention trail using low fat and restricted calories during the first transplant year, nutrition and body composition improved as demonstrated through decreased body fat, weight loss, lower serum cholesterol, improved fasting glucose, and increase serum albumin (69). In this trial, dietary compliance was better in man, in whom greater losses in fat mass where displayed than in female study subjects. Since it has been suggested that by some groups that the consequences of the metabolic syndrome have a lasting and profound effect on allograft prognosis within the first post-transplant year, intervention designed to begin within 3 months of engraftment must be considered with longer durations of the patient follow up. In contrast malnutrition in renal transplant recipient was the subject of detailed pre-transplant and post-transplant nutritional assessment in the group of diabetic and non-diabetic renal failure patients. These subjects displayed multiple nutritional markers indicating that they fall with the bottom 5.0 percentile of PCM.

After completing a subjective global assessment, comprehensive nutritional counseling and meal planning were instituted post transplantation. Surprisingly this nutritional intervention led to improvement in 42% of diabetics and 29% of non-diabetics. The patients showed significant improvement in weight gain, triceps skinfold thickness, mid-arm muscle circumference (MAMC), and serum albumin and transferring (70).

### Different risk factor effecting nutrition in ESRD

#### Education and understanding

Study performed by Vergili and Wolf evaluating the renal services performed by doing the survey about renal dietitians practices done in HD centers indicated substantial disparity in renal dietitians practice and Kidney Disease Quality Outcomes Initiatives. The survey queried dietitians about healthy body management, nutritional indicators, fluid management, metabolic parameters, adjusted body weight, counseling and many more. Outcome of the study was that all dietitians agreed to have new guidelines for dietary interventions. The response rate of the study was 68.3% (10,71). An improved understanding of the implications of kidney disease and variable risk factors such as obesity, smoking, hypertension will support the behavioral and lifestyle changes that can deliver the preventative dividend (72). A systematic review, carried out in the United States showed that the patients of ESRDs were mostly old aged. They were at high risk because of the lower immune status. Due to which they were always exposed to multiple infections during dialysis. These infections during dialysis were preventable by achievement of phosphate control, treating anemia effectively before dialysis, correction of puff serum albumin levels and use of fewer intravascular catheter and also optimizations of dialysis dose. This study described that by improving different services; the chances of risk factors can be decreased. Which were procedural, for example, implementation patterns of policies in a facility, attitudinal, for example, staff morale, personal, for example, staff communication, belief orientation, for example, by giving the clear concept of dialysis and other diseases and making them clear according to their religious views as indicated in clinical performance measured (CPM) (73).

#### Late referrals

Study performed by the Hussaini et al indicated that nephrologist should follow patients of RRT from their first visit at renal unit (53**)**. A systematic review carried out in the United States showed that late referral of a patient with CKD to nephrologists is associated with the poor clinical outcome. Longer pre-dialysis care by nephrologists may result in a reduction in rates of the hospitalization. There is some key factor, which is associated with the late referral of the patient to the nephrologists that are being older, belonging to the minority group, being less educated, being uninsured and suffering from co morbidities (hypertension, diabetes, CVD, etc.) and lack of communication between primary care physicians and nephrologists contribute to the late referral (74). Campbell et al found that more than 90% of referring physicians felt that they had inadequate training timing or indication for the referral of the patient with CKD (75,76). Gordon et al showed that primary care physicians identified patient with CKD later, performed lesser diagnostic work up, and statistically significantly less likely than Black race was not associated with the late referral due to the their ability to adjust with co-morbidities and multiple demographic factors and status (76,77). Geographic difference in practice pattern may also explain some of these racial differences in the propensity to refer blacks and whites to nephrologists for renal replacement therapy. Geographic difference in practice pattern may also explain some of these racial differences in the propensity to refer blacks and whites to nephrologists for renal replacement therapy (77).

#### Mistakes in prescription

Related articles also highlight the facts that the prescriptions of the doctors for drug also play an important role in the effectiveness of the dietary intervention on pathogenesis of the CKD. The research work done on prescription of doctor’s shows that there are a lot of omissions and error in the hospital prescriptions. According to the survey over 23.9% of prescriptions were illegible and 29.9% of the prescriptions were incomplete. Legibility and completeness are higher in unusual drug prescription. The number of wrong prescription illegibility above 20% is the unacceptability high. Values need to be improved by enhancing the safety culture and in particular the awareness of the professionals on the consequence that bad prescription writing can produce (78). Ridley states that almost 50% of all prescription error in the intensive care unit is due to four categories: not writhing the order according to the formulary, ambiguous medications order, none standard nomenclature, and writing illegibility.

Since there are multiple risk factors, which are associated with the a large proportion of wrong medication prescription, an intervention is needed to enhance the safety culture in all settings by improving clinical documentation and through enhanced the professional awareness of potential medications errors related to bad prescription writing. This is a problem of world’s different hospital (79).

#### Low immune status and lack of donors

A literature review performed by Plourd, to assess the nutritional management in ESRD patients with AIDS. He indicated that AIDS is also co-existing condition as other co morbid condition like hypertension and CVD in ESRD patients. He showed that the development of malnutrition in AIDS population is due to multifactorial as in development of malnutrition in ESRD populations such as drug-drug interaction, alcohol and drug abuse, malabsorption and diarrhea, low calorie diet or unpalatable diet, decreased physical activity and many more. Additional research is necessary to define in the nutritional needs and management of this high-nutritional risk population (80). In the United States, it has been clear for some years that early acute rejection and long term graft survival are worse in patients of African-American ethnicity than of white ethnicity. The reasons for this seem to include an increased immune responsiveness, partly explained by worse human leukocyte antigen (HLA) matching between donor and recipient, and also worse outcomes associated with social status (81). In the United Kingdom, graft survival is also worse in those of black ethnicity compared to those of white ethnicity, but those of south Asian ethnicity do not seem to be disadvantaged (82). Quality of life is also linked to ethnicity, but this may be associated with the social status of a particular ethnic group, rather than differences in the outcome of a transplant (83). Other complications of transplantation are associated with ethnicity, especially new onset diabetes after transplantation, which is more frequent in those of black and south Asian ethnicity (82,83). In short, as with other areas of society and medicine, ethnicity is an important factor impacting on the practice of renal transplantation, with both cultural and biological differences between ethnic groups affecting access to, and the results of renal transplantation.

#### Poor diagnostic methods

Motokawa et al performed a research process in which they try to check the nutritional status in CKD patients on dialysis by using subjective global assessment (SGA) method. They also compared SGA with total body potassium (TBK), considered as golden standard method for measuring body cell mass (BCM). SGA is a clinical assessment tool, is just one of panel of nutritional indictor recommended by the National Kidney Foundation Kidney disease Outcomes Quality Initiatives. It is based on clinical parameters such as medical history (weight change, dietary intake change, gastrointestinal symptoms, and changes in functional capacity) and physical examination (assessment of subcutaneous fat and muscle mass store). SGA is completely based on the clinical experience of medical professionals. A potential benefit of SGA is such tool is that degree of malnutrition can be easily detected. The study indicated that it is the most appropriate method to measure the malnutrition in pre dialysis patients. It is cheap also as compared to other laboratory procedure available in market (85). On the other hand, albumin is considered as the potential tool for measurement of malnutrition in patients on RRT. Lacson et al performed the study to estimate the effect of an improvement in nutrition, represented by albumin concentration on hospitalization, mortality and Medicare ESRD program cost. The study indicated that nutritional intervention that increases serum albumin through oral nutritional supplements could lead to good clinical outcome. It will also affect the mortality rate and bring down the treatment cost of ESRD patients (6,84). On the other hand, there some studies which refused the authenticity of albumin as well as creatinine in determining the function of kidneys. A study was performed in Sal ford, United Kingdom, to study the relationship between prevalence of CKD in the DM and to examine the ability of keratinize and albumin urea to early detection of the CKD which was used in the laboratory as a standard parameter in the early detection of CKD at the primary care level. Results showed the failure of serum creatinine and albumin urea in early detection for the CKD. The other proposed method is early screening. Screening has been effective at some spots but still in need of a lot of improvements (85). Historically CKD has been a grossly under diagnosed condition, however many patients now being diagnosed with CKD would have otherwise ended up needing emergency dialysis in years to come, without any previous diagnosis. Dialysis cost per patient is £20 000 to £25 000. Early detection is cost effective, in both financial and human terms. Therefore, the attention must be paid to develop strategies for early detection of CKD to prevent serious complication and save money.

#### Summary

The findings suggest that the diet in ESRD can be impaired by multiple co factors that make the situation worse. As health care resources are scarce, understanding its prevalence, disease awareness and identification of the early risk factors of CKD is mandatory and beneficial in determining the most cost effective strategies for the prevention of ESRD.

## Limitation of the study

- The time duration for carrying out systematic review was really short. Most of time was utilized for retrieving article due to several protocol involved. It was bit hard to do all alone. However, we tried to complete within a short time period (3-4 months).
- The research was limited by the lack of approach to get access to the large number of article on dietary effects that are put as intervention to stop the pathogenesis of ESRD. Therefore, the outcome of research would have been better if there was possible approach or access to the articles.
- The research was self-sponsored. Another big hurdles was the lack of funds to obtain the original articles. However, the University of Brighton library staff helped me in obtaining the articles used to complete research work.

## Strength of study

- It consists of different research design that is RCT, cross sectional studies, literature reviews and national surveys.
- Research articles selected were from different environment including Japan, the United Kingdom and the United States assessing variable factors effecting diets. The results of the study indicated multiple factors that are affecting outcome of good diet on the pathogenesis of ESRD.
- Moreover, study highlighted boarder range of nutritional practices performed by renal dietitian and health professionals than previous published studies of which we were aware, and compared those practices with a wide range of references.

## Target for future research

- A project improvement in the nutritional state represented by biomarkers such as serum albumin provides a reasonable likelihood for intervention to reduce death rate, hospitalization events and ESRD related cost.
- Practices in field of renal nutrition must have the ability to assess and intervene so that these patients are able to attain or maintain the best nutritional status possible.
- The underlying causes of the fractional synthesis of many proteins in muscle also need attention. The results for of these studies may substantially contribute to improvement in the treatment of patients with chronic renal patients.
- The development of new practice guidelines, particularly for fluid management, would be appreciated by renal dietitians, and appear to be warranted.
- This disparity may be related to disbelief that current clinical practice guideline on weight and energy requirement are not valid and are in need of revision. Practices related to the metabolic monitoring are, on the other hand, quiet congruent with KDOQL guidelines.
- Special attention needs to be focused on the additional impact of immunosuppressive medications and as to how steroids withdrawal may be accelerated with the advent of steroids sparing immunosuppressive regimens.
- Clinical research efforts using interventional dietary trials for multiple post-transplant complications, especially obesity and metabolic syndrome, have been lacking and are urgently needed.
- A major effort to improve the long-term nutritional plight of lung transplant recipients should use interventions that contribute to improve PCM, reduce on going chemokine’s mediated inflammation and limit the impact for post-transplant metabolic syndrome.

## Conclusion

The research work indicated diet in ESRD patients is a complex topic and there are multiple associated factors that stop the potential effect of diet. ESRD disease is always associated with many co-morbid conditions that made the scenario worse. The risk factors are listed in Table 1.

**Table 1:** Factors influencing nutritional status

**Physical**	**Environmental**	**Psychological**
Anorexia related to uremia, fever, or treatments Malabsorption and diarrhea, Nausea and vomiting related to chemotherapy, gastritis, esophagitis, reduced peristalsis, Low calorie or palatable diet, altered mental status related to dementia, Drug nutrients interaction, decreased physical activity	Poverty Inadequate cooking facilities Social isolation Unacceptable or unavailable meal delivery program Culturally preferred food unavailable Unsuitable housing Inadequate knowledge and education	Depression Desire for death Food aversions related to alteration in taste, texture, and smell Altered perception of food value: fat diet, junk food, mega dosing nutrition’s, using special food. Eating non-food substances Fear of somatic problems leading to anticipatory nausea and vomiting or fear of diarrhea Individual dietary hobbits Unable to take medications are prescribed

CVD and CKD are common comorbid conditions. Life style, particularly diet is a critical component of treatment for these conditions. Register dietitians play a key role in bridging the gap between the science of nutrition and the empowerment of individuals to alter their lifestyles in a healthy manner. A range of dietary manipulations has been reported to reduce risk factors and decrease risk of CVD and CKD outcomes. However, many studies provided food to participants or were limited to adjustment of few specific nutrients. Diet intervention in relation with ESRD is really complicated topic. As multiple co-morbid conditions such as hypertension, CVD, CKD, and DM are associated with ESRD, which made the scenario really worse while fixing the dose of any diet. Still a lot of research work is required to understand this topic.

## Authors’ contribution

SM, HA and AA wrote the review and completed the draft. BMM, KTB and CMJN edited and completed the final manuscript.

## Conflicts of interest

Authors declared no conflicts of interest.

## Ethical considerations

Ethical issues (including plagiarism, data fabrication, and duplicate publication) have been completely observed by the authors.

## Funding/Support

None.
